# Mesenchymal Stem Cells as Promoters, Enhancers, and Playmakers of the Translational Regenerative Medicine 2018

**DOI:** 10.1155/2018/6927401

**Published:** 2018-10-30

**Authors:** Andrea Ballini, Stefania Cantore, Salvatore Scacco, Dario Coletti, Marco Tatullo

**Affiliations:** ^1^Department of Basic Medical Sciences, Neurosciences and Sense Organs, University of Bari “Aldo Moro”, Bari, Italy; ^2^City Unity College, Athens, Greece; ^3^Institut de Biologie-Biology of Adaptation and Aging (B2A), Université Pierre et Marie Curie, Paris, France; ^4^Biomedical Section, Tecnologica Research Institute, Crotone, Italy

Mesenchymal stem cells (MSCs) are currently being tested in preclinical and clinical trials for their ability to foster wound healing and tissue regeneration [1].

They are well known to show a therapeutic potential largely depending on their ability to secrete proregenerative cytokines, making these cells an attractive option for improving the treatment of chronic wounds. The wound microenvironment is a miscellaneous key factor in the local management of the healing process: players such as the extracellular matrix or the resident and recruited cells with paracrine activity are able to determine the way and the appropriateness of the regenerative processes [2].

Dental-derived mesenchymal stem cells (D-dMSCs) are an intriguing milestone of the regenerative medicine, with regard to their potential of differentiating into osteogenic, adipogenic, and chondrogenic lineages [3–5], possessing in this way the potential to significantly influence the bone and periodontal treatment strategies in the future [6–9].

Despite the multiple barriers to their clinical use, MSCs or D-dMSCs have shown sufficient promise to garner a primary place in the field of translational medicine. In fact, MSC and D-dMSC therapies have significant implications for human health: clinical studies are greatly needed to confirm or stimulate the basic and translational researches aimed at reaching cutting-edge results [10–13].

The special issue has reported articles on MSCs used as a therapeutic aid in clinical and surgical applications. The topics in translational medicine reported were the MSC therapy for intravertebral disc regeneration (J. Jia et al.) and the cell therapy as a promising aid for cerebral vasculature (B. Y. Choi et al.), as well as for the proper management of thin endometrium (J. Zhao et al.).

The most reported translational use of MSC/D-dMSC therapy is related to bone tissue regeneration: in fact, many authors have investigated on the osteogenic ability of different stem cell types and genes, such as TGF*β*1 that enhances MSC commitment to either the osteogenic or adipogenic lineages by reorganizing the actin cytoskeleton (M. Elsafadi et al.), as well as on the use of a PRP blood clot stabilizer to treat infrabony periodontal defects (M. Saleem et al.) and the use of vitamin D in dental-derived MSCs that promote osteogenic differentiation through the modulation of *α*V*β*3 (F. Posa et al.), the role played by the ganglioside GM1 in the osteogenic differentiation of human tendon stem cells (S. Bergante et al.), or via low-frequency pulsed electromagnetic fields (P. S. P. Poh et al.)

Some authors have focused their researches on umbilical cord stem cells, due to their large application on translational medicine (D. R. Kwon et al.), as well on miRNA-132 MSC-derived exosomes in the treatment of myocardial infarction (T. Ma et al.).

Finally, experimental findings from in silico studies, on the one hand, highlighted the promotive role of hypoxia in MSC proliferation (S. Gao et al.); on the other hand, it was reported that an *in vitro* loading model (2D and 3D in combination with different scaffolds) represents a simple and very efficient way to investigate molecular events during orthodontic tooth movement (M. Janjic et al.).

In this special issue, the editors together with the involved authors have well described the MSCs and D-dMSCs in their different but fundamental roles as promoters, enhancers, and playmakers of the translational regenerative medicine. Starting from the contents of our issue, the scientific community will be stimulated to experiment new ideas, to improve the knowledge of the MSCs/D-dMSCs, and to speed up their clinical application, so as to improve the future therapies.
